# Supplementary material for: Machine Learning Model in Predicting Sarcopenia in Crohn’s Disease Based on Simple Clinical and Anthropometric Measures
Source: Int J Environ Res Public Health. 2022 Dec 30;20(1):656. doi: 10.3390/ijerph20010656 (PMC9819919; doi:10.3390/ijerph20010656)
Supplement: Supplementary file 1 [file ijerph-20-00656-s001.zip › ijerph-2020535-supplementary.pdf]

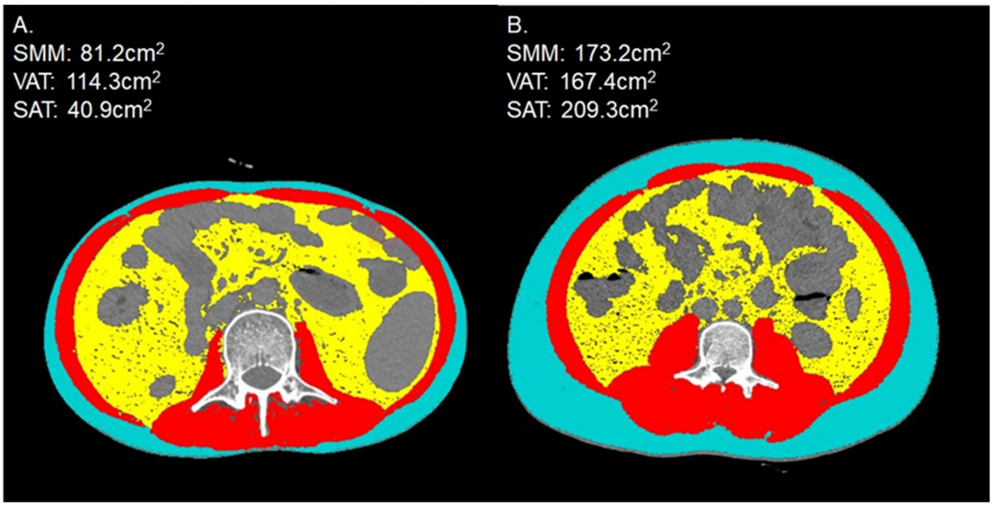

Figure S1. L3 segmentation and calculation of body composition index in a sarcopenic and non-sarcopenic patient.

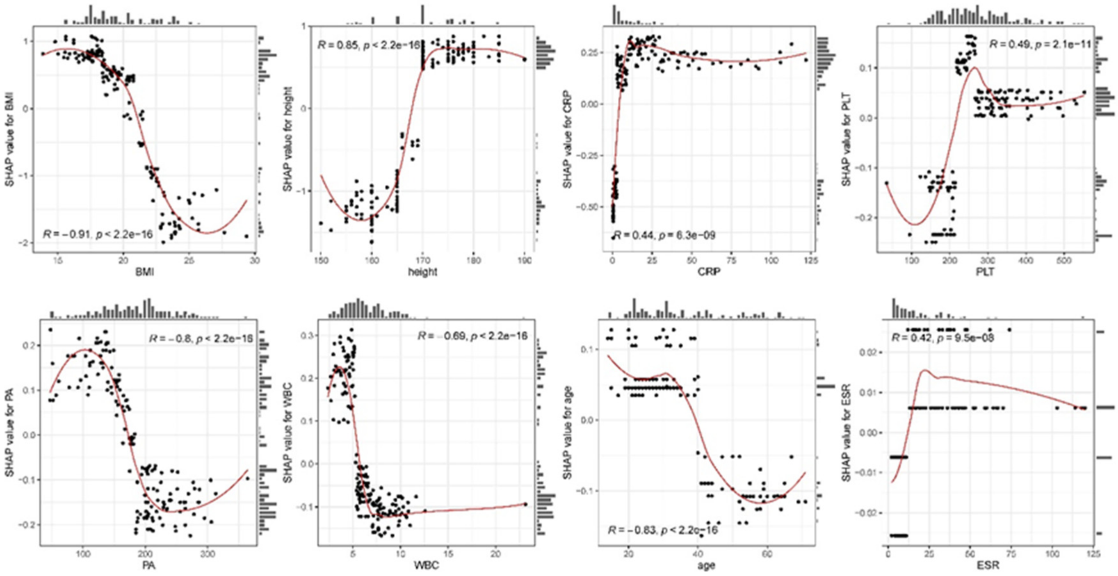

Figure S2. SHAP dependence plot of the LightGBM model. Dependence plot depicts how each variable affects the prediction of sarcopenia.

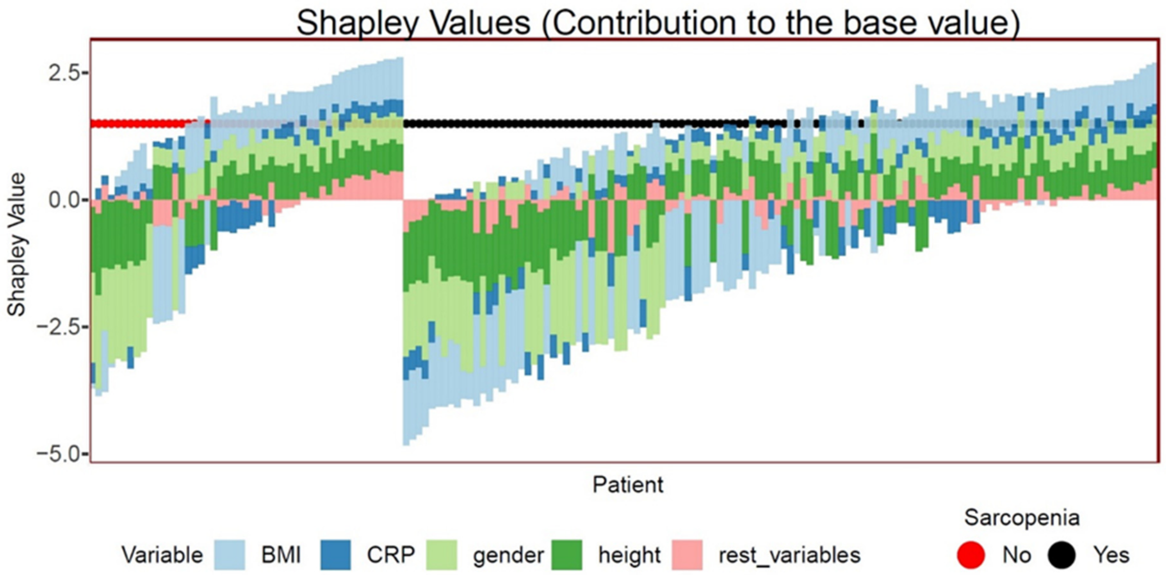

Figure S3. SHAP force plot of the LightGBM model. Each stacked bar represents one patient and their total Shapley score. The predicted sarcopenic state of each patient is determined by his/her total Shapley value.
